# Mating Frequencies of Honey Bee Queens (*Apis mellifera* L.) in a Population of Feral Colonies in the Northeastern United States

**DOI:** 10.1371/journal.pone.0118734

**Published:** 2015-03-16

**Authors:** David R. Tarpy, Deborah A. Delaney, Thomas D. Seeley

**Affiliations:** 1 Department of Entomology, North Carolina State University, Raleigh, North Carolina, United States of America; 2 W. M. Keck Center for Behavioral Biology, North Carolina State University, Raleigh, North Carolina, United States of America; 3 Department of Entomology and Wildlife Biology, University of Delaware, Newark, Delaware, United States of America; 4 Department of Neurobiology and Behavior, Cornell University, Ithaca, New York, United States of America; San Diego, UNITED STATES

## Abstract

Across their introduced range in North America, populations of feral honey bee (*Apis mellifera* L.) colonies have supposedly declined in recent decades as a result of exotic parasites, most notably the ectoparasitic mite *Varroa destructor*. Nonetheless, recent studies have documented several wild populations of colonies that have persisted. The extreme polyandry of honey bee queens—and the increased intracolony genetic diversity it confers—has been attributed, in part, to improved disease resistance and may be a factor in the survival of these populations of feral colonies. We estimated the mating frequencies of queens in feral colonies in the Arnot Forest in New York State to determine if the level of polyandry of these queens is especially high and so might contribute to their survival success. We genotyped the worker offspring from 10 feral colonies in the Arnot Forest of upstate New York, as well as those from 20 managed colonies closest to this forest. We found no significant differences in mean mating frequency between the feral and managed queens, suggesting that queens in the remote, low-density population of colonies in the Arnot Forest are neither mate-limited nor adapted to mate at an especially high frequency. These findings support the hypothesis that the hyperpolyandry of honey bees has been shaped on an evolutionary timescale rather than on an ecological one.

## Introduction

The Western honey bee (*Apis mellifera* L.) is incredibly widespread and ecologically successful, as its native range spans all of Africa, Europe, and much of Western Asia [1]. Aside from the now-extinct *Apis nearctica* [2], honey bees are an exotic species in North America, having been introduced by European settlers beginning early in the 17th century. These bees quickly escaped from beekeepers’ hives and spread throughout North America, creating two groups of honey bee colonies: managed colonies living under the supervision of beekeepers in apiaries and feral colonies living on their own in the wild.

In recent decades, many populations of honey bees have experienced unusually high mortality as a consequence of various factors (reviewed in [3]). Research on the possible causes of this mortality has focused on parasites and pathogens, nutritional stress, habitat loss, and toxic and sub-lethal levels of pesticides [4,5,6,7]. One major cause of colony mortality in Europe and North America is the ectoparasitic mite *Varroa destructor*, which was introduced to these continents in the 1970s and mid 1980s, respectively. Conventional wisdom holds that, if a colony of honey bees in the managed population does not receive mite control treatments, the mites (vectoring several associated viruses) will kill the colony within 2 to 4 years [8,9]. The introduction of *Varroa* is widely thought to have decimated the wild colonies in Europe and the feral population in North America [10,11]. However, the increasing reports of feral honey bee colonies surviving in several eastern and southern states in the U.S. [12]—as well as the persistence since at least the 1970s of a population of feral honey bee colonies in the Arnot Forest in New York State [13]—indicate that feral honey bees in North America have survived and may even be rebounding despite the presence of *Varroa* and other disease agents.

How honey bee colonies tolerate pathogens and parasites has been the focus of many studies, and special attention has been paid to several behavioral mechanisms, termed ‘social immunity’ [14,15], that are inherent to many insect societies. One such mechanism is female multiple mating, or polyandry, which is taxonomically restricted among social insects [16] but occurs at an exceptionally high level in honey bees (genus *Apis*). Estimates of the mating number of *Apis mellifera* queens, based on molecular genotyping of worker offspring, vary between 1 and 59 and average about 14 [17]. Many hypotheses have been proposed to explain why this hyperpolyandry has evolved in honey bees (reviewed by [18]), and the “intracolonial genetic diversity” hypotheses are widely seen as the most plausible [19]. While the benefits of increased intracolonial genetic diversity include an increased behavioral repertoire of the worker force (e.g., [20]), and reduced variation in diploid male production as a consequence of the single-locus sex determination system (e.g., [21]), they also include a lower likelihood of devastating infestations of parasites and pathogens among colony members [22,23,24]. One key to the success of feral honey bee colonies may be, therefore, that their queens have an extremely high mean mating frequency, so their colonies possess unusually high genetic diversity and have exceptional ability to tolerate pathogens and parasites.

The aim of this study was to examine the mating frequencies of the queen bees in a remote and persistent population of feral honey bee colonies in rural New York State. We compared the mating frequencies of queens in these feral colonies to those in the nearest managed colonies to see if mating frequency has been shaped by ecological context. One possibility is that queens in a feral population, where colonies are widely spaced, may have lower mating frequencies because of the lower colony density and diminished availability of mates. If so, then this will suggest that their survival depends upon something other than extreme polyandry. An alternative possibility is that queens in a population of feral colonies may have higher mating frequencies. If so, then they might have increased benefits of intracolony genetic diversity which might explain why they have been able to persist despite *Varroa* and other disease agents. The null hypothesis, of course, is that queens in feral colonies and managed colonies have the same mating frequencies, which would suggest that the polyandry of queen honey bees has been shaped by natural selection acting on an evolutionary timescale and has not been tuned on an ecological time scale through intense selection recently for greater resistance to parasites and pathogens.

## Materials and Methods

**Fig 1:**
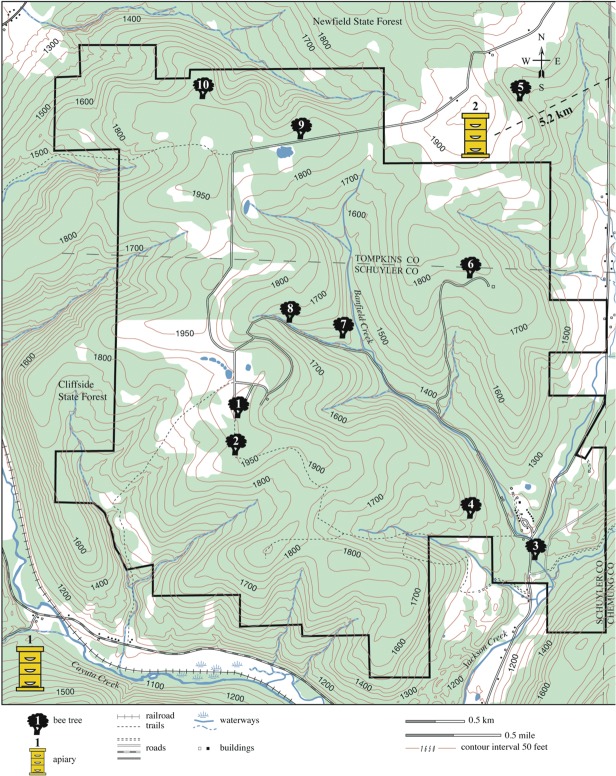
Study population. Map depicting the locations of the honey bee colonies that were investigated in this study. Shown are the locations of the 10 wild colonies of honey bees (bee trees) in the Arnot Forest, and of the two nearest apiaries outside the Arnot Forest.

The Arnot Forest (Fig. 1) is a 17-km^2^ research preserve owned by Cornell University and located approximately 25 km south of Ithaca, in New York State (42° 17’N, 76° 39 W). It is home to a population of feral honey bee colonies that has been studied since the 1970s. The first census and mapping of this population was performed in 1978 [25]. This work was repeated in 2002, some 15 years after the arrival of *Varroa destructor*, confirming the continued existence of the honey bees living in the forest [13]. In August 2011, we built on this prior work by locating approximately half the colonies in the Arnot Forest and the surrounding state forest lands (an area of some 25 km^2^, total) using the same technique (‘bee-lining’; see [26] that one of the authors (TDS) used in 1978 and 2002). Consistent with the density of feral colonies found previously in this forest area—about one colony per km^2^—we located 10 feral colonies living in trees within or just outside the Arnot Forest (see Fig. 1).

We sampled workers from each feral colony by capturing them from the feeding station when it was moved to within 100 m of the tree that the colony occupied, at which point the feeding station was 1000s of meters from any competing colonies and so was completely dominated by foragers from the nearby colony (as confirmed by separate genetic analyses, i.e. Seeley et al., in press). More than 100 foragers visiting the feeder were collected, stored, and preserved in 95% ethanol. We also sampled ∼100 workers from each of 20 managed colonies (i.e., 10 colonies per apiary in the two nearest apiaries located outside of the Arnot Forest; these apiaries contained 22 and 24 colonies, respectively). Apiary 1 was 1.0 km from the southwest boundary of the Forest and Apiary 2 was 5.2 km from the northwest boundary (see Fig. 1). These were the only managed colonies found within 6 km (the typical flight range of honey bees) of the Arnot Forest and were used to provide information on the mating frequency of commercially produced queens. Apiary 1 was started in April 2011 with mated queens newly purchased from Wooten’s Golden Queens, Palo Cedro, California. Apiary 2 was established in the early 2000s and its colonies received, from time to time, new queens purchased from Wooten’s Golden Queens.

After collecting the adult worker bees from each feral and managed colony, we individually extracted their DNA following the Chelex method (see [27]). We then analyzed each bee at 12 microsatellite loci (A24, A28, A79, A88, A107, Ap43, Ap66, Ap81, AC006, B124, HB-THE-03, and HB-THE-04). One multiplex PCR was used to amplify 10 μl reactions of extracted DNA containing 1.0–4.0 mM of fluorescent dye-labeled primer (Applied Biosystems), 1x reaction buffer, 1.5 units *Taq* polymerase, 3mM dNTP mixture, 0.001 mg bovine serum albumin, 1 μl DNA, and 25 mM MgCl_2_. We ran the PCR products from all individuals on a Perkin-Elmer ABI Prism 3730XL automated capillary DNA sequencer and determined paternal inheritance with GeneMapper 4.0 software. This practice has become standard across most studies investigating mating number (more precisely, the effective paternity frequency of the workers) by queens and the resultant genetic structure of honey bee colonies (e.g., [28,29,30,31,32]).

We tabulated a paternal marker set for each worker to estimate the genetic structure within each colony using COLONY 1.2 [33]. The total number of different marker sets within a colony signified the observed paternity frequency of the queen (*N*_*o*_), or the total number of drone fathers that are represented in the offspring. We also determined the proportion of each subfamily within a colony so that we could calculate the effective paternity frequency (*m*_*e*_) using the sample statistic proposed by Nielsen et al. [34],

$$m_{e}=\frac{{\left(n-1\right)}^{2}}{\sum_{i=1}^{N_{o}}p_{i}^{2}\left(n+1\right)\left(n-2\right)+3-n}$$

where *n* is the number of worker samples and *p*_*i*_ is the proportion of subfamily *i* within the sample population. This unbiased measure is used for calculating skews in paternity and intracolony genetic relatedness.

We then compared the mating frequencies across the three groups of colonies (Apiary 1, Apiary 2, and feral colonies) using analysis of variance. We used Tukey post-hoc comparisons to identify differences among the means of the three groups of colonies with respect to observed and effective mating numbers. All statistical tests are two-tailed with α = 0.05, and all means are reported as ± SD unless otherwise specified.

We entered the drone alleles from each population into the microsatellite toolkit 3.1.1 in Excel [35] using the haploid data option, from which we determined the allelic frequencies, sample size, and the mean number of alleles by population. We calculated drone diversity by population using Nei’s unbiased gene diversity.

## Results

We analyzed 1,089 worker bees, hence 36.2 ± 8.35 individuals per colony (range 13–47). We excluded microsatellite loci that produced consistent PCR products in fewer than 25% of the individuals from a given colony. Interestingly, there was an unusually high frequency of null alleles in all three groups for several of the microsatellite loci, and these loci were also excluded to avoid potential scoring errors and inflated estimates of effective paternity. Nonetheless, most colonies were analyzed with 6–10 loci, each with high allelic variability, so that the average non-detection error [36] was extremely low (2.49x10^−11^).

**Fig 2:**
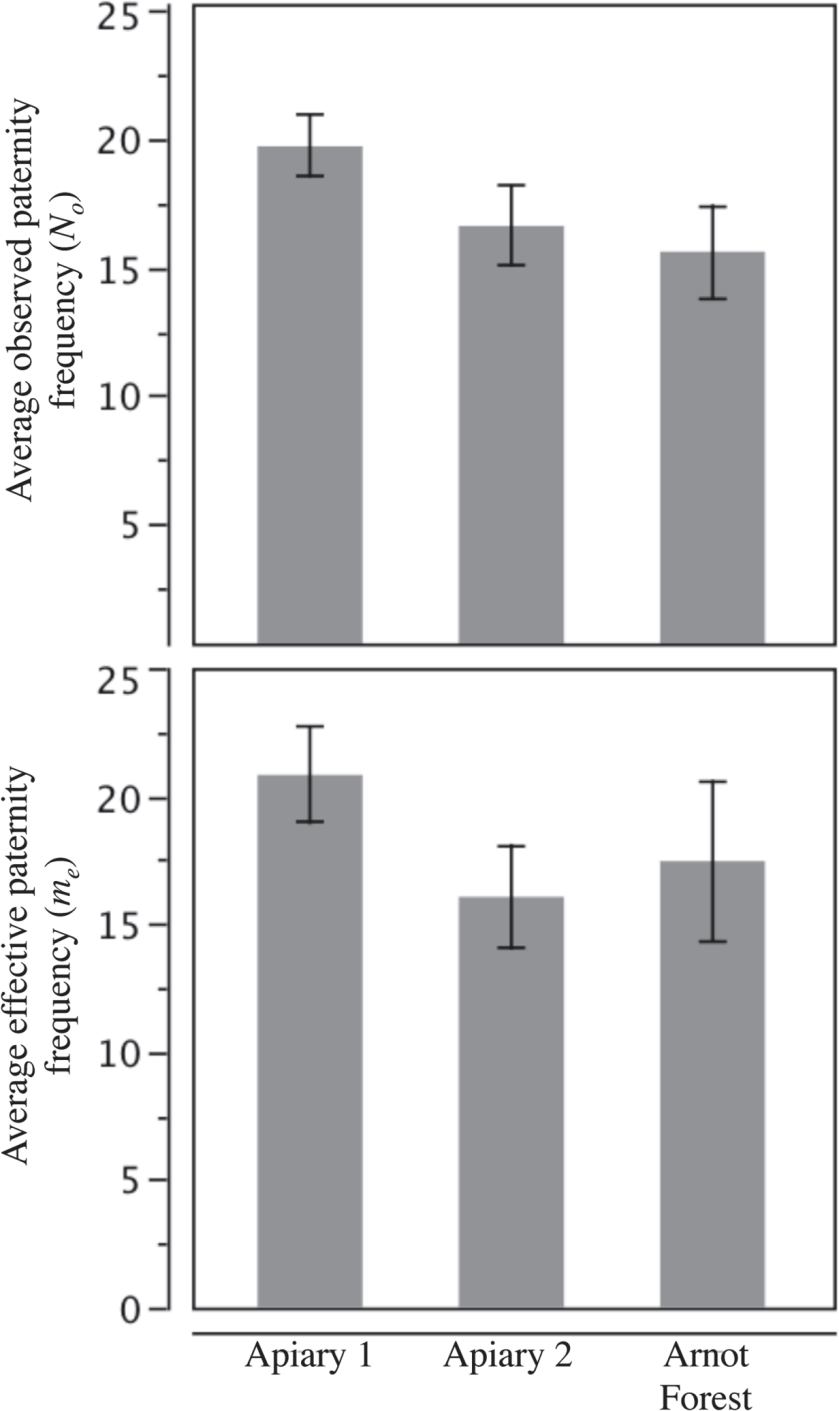
Mating frequencies of queens from feral and managed colonies. Average ± SEM of the observed (top) and effective (bottom) mating frequencies of the 10 honey bee queens in each of the three groups of colonies. No significant differences (at α = 0.05) were found among the three groups.

We found no significant differences in mean mating frequency among the three groups (Fig. 2). The mean effective mating frequencies (*m*_*e*_) of the Apiary 1 queens (20.8 ± 5.9) and the Apiary 2 queens (16.0 ± 6.4) were not statistically distinguishable from that of the Arnot Forest queens (17.4 ± 9.8; *F*_*2*,*27*_ = 1.04, P = 0.36). Moreover, all three estimates lie well within the range of mating frequencies reported previously for *Apis mellifera* queens [17,31].

We obtained genotypic data for 574 drone fathers total among the three groups (Table 1). The unbiased gene diversity was highest in drones from Apiary 1 (0.76 ± 0.03) followed by the drones from the Arnot Forest (0.73 ± 0.04) and the drones from Apiary 2 (0.61 ± 0.07). The mean numbers of alleles per locus for drones from Apiary 1, the Arnot Forest, and Apiary 2 were 9.90 ± 4.77, 9.70 ± 3.47, and 8.10 ± 4.36, respectively. Closer inspection of the allele frequencies of drone fathers from the different populations (Table 2) reveals that the number of unique alleles (i.e., those not found in either other population) follows a similar pattern. Apiary 1 had 1–7 unique drone alleles in eight of the 10 loci analyzed (HB-THE-03, A107, A113, AC006, A28, AP66, AP81, and B124), Apiary 2 had 1–5 unique drone alleles in 5 of the 10 loci analyzed, and the Arnot Forest was intermediate with 1–9 unique drone alleles in 7 of the 10 loci analyzed (Table 2).

**Table 1:** Population statistics for drone fathers.

**Population**	**Sample Size**	**Unbiased Gene Diversity and SD**	**Mean No. of Alleles and SD**
Arnot Forest	156	0.73 ± 0.04	9.90 ± 4.77
Apiary 1	197	0.76 ± 0.03	9.70 ± 3.47
Apiary 2	166	0.61 ± 0.07	8.10 ± 4.36

Population genetics statistics for drone fathers from the Arnot Forest, Apiary 1, and Apiary 2, based on alleles from 10 variable microsatellite loci. Drone alleles inferred from worker and queen genotypes using the program COLONY 1.2 [33].

**Table 2:** Alleic frequencies of drone fathers.

***Locus HB-TDE-03***				***Locus 107***				***Locus A113***			
	**Arnot Forest**	**Apiary 1**	**Apiary 2**		**Arnot Forest**	**Apiary 1**	**Apiary 2**		**Arnot Forest**	**Apiary 1**	**Apiary 2**
***104***	1.03			***144***		3.13		***146***		15.43	
***144***		4.00		***152***	9.09			***148***		1.14	
***146***	1.03			***154***	3.64		0.91	***154***		0.57	
***156***	6.19			***156***	3.64		1.36	***166***			0.53
***158***	3.09			***158***	1.82	5.21	4.09	***176***			0.53
***160***	2.06			***160***	10.91		3.64	***208***	4.00		1.59
***162***	10.31	13.00		***162***	36.36	8.33	15.91	***214***	28.00	14.86	32.28
***164***	4.12			***164***	5.45	2.08	10.00	***216***	4.00	4.00	3.17
***166***	1.03	1.00		***166***	1.82	3.13	4.09	***220***	39.33	40.57	50.26
***168***	7.22	5.00		***168***	1.82	20.83	16.36	***222***	4.00	8.00	1.06
***172***	4.12			***170***	3.64	7.29	4.09	***226***	12.67	8.00	4.23
***178***		5.00		***172***	5.45	1.04	11.82	***228***	6.00	3.43	5.29
***180***	1.03			***174***			0.91	***230***	2.00	4.00	
***182***	7.22	6.00	3.57	***176***	1.82		2.27	***234***			1.06
***184***	1.03	1.00	12.50	***178***	12.73	6.25	8.64				
***186***		1.00		***180***			2.73				
***188***	4.12		2.68	***182***	1.82						
***190***	1.03	1.00		***183***		1.04					
***192***	4.12	24.00	11.61	***184***		11.46	11.82				
***194***	31.96	32.00	51.79	***196***			0.45				
***196***	4.12	7.00	5.36	***198***			0.45				
***198***	2.06		10.71	***214***		3.13					
***200***	2.06		1.79	***216***		6.25					
***220***	1.03			***220***		10.42					
				***222***		5.21					
				***228***		5.21					
***Locus AC006***				***Locus A024***				***Locus A28***			
	**Arnot Forest**	**Apiary 1**	**Apiary 2**		**Arnot Forest**	**Apiary 1**	**Apiary 2**		**Arnot Forest**	**Apiary 1**	**Apiary 2**
***100***		0.58		***92***	10.64	12.90	13.55	***92***		6.09	
***102***		1.73		***94***	3.19	9.68	25.81	***94***		2.61	
***104***		12.72		***96***	2.13			***100***		0.87	
***146***	46.43	49.13	58.80	***98***	1.06			***102***		12.17	
***148***	17.26	2.89	6.48	***100***			4.52	***104***		25.22	
***150***	3.57		3.70	***102***	17.02	4.84	15.48	***106***		1.74	
***154***	11.90	20.81	14.81	***104***	19.15	37.10	35.48	***126***	0.63		
***156***	17.86	9.25	13.43	***106***	6.38		3.87	***128***	6.96	11.30	17.03
***158***	2.38	2.89	0.93	***128***	7.45			***132***	0.63		
***160***			0.46	***134***	32.98	35.48		***134***	74.05	40.00	82.97
***164***			0.46	***146***			1.29	***136***	3.16		
***168***	0.60							***140***	1.90		
***214***			0.46					***144***	6.33		
***220***			0.46					***150***	1.90		
								***152***	4.43		
***Locus A88***				***Locus AP66***				***Locus AP81***			
	**Arnot Forest**	**Apiary 1**	**Apiary 2**		**Arnot Forest**	**Apiary 1**	**Apiary 2**		**Arnot forest**	**Apiary 1**	**Apiary 2**
***128***	3.15	6.99		***91***	29.03	4.65	87.21	***93***		2.76	
***132***	2.36			***93***	41.94	41.86	4.65	***124***	5.56	2.76	
***134***	19.69	42.66	6.50	***96***	12.90	1.55	6.98	***126***	9.26	34.48	16.20
***136***	43.31	21.68	41.46	***99***	16.13	1.55	1.16	***134***	64.20	52.41	73.74
***140***	2.36	10.49	10.57	***101***		0.78		***135***		0.69	
***142***	3.15			***134***		4.65		***136***	12.35	1.38	7.82
***144***	13.39	14.69	29.27	***136***		17.83		***138***	7.41	4.83	2.23
***146***	1.57		2.44	***140***		2.33		***150***	1.23	0.69	
***150***	1.57	0.70	0.81	***142***		3.88					
***152***	9.45	2.80	8.94	***144***		17.05					
				***146***		0.78					
				***150***		3.10					
***Locus B124***	**Arnot Forest**	**Apiary 1**	**Apiary 2**								
***134***		2.80									
***214***	5.13										
***216***	29.49	39.16	42.00								
***218***	10.26	17.48	12.00								
***220***	5.13	9.79	10.00								
***222***	8.97	3.50	9.33								
***224***	20.51	14.69	8.67								
***226***	5.13	8.39	4.67								
***230***	2.56	2.10	10.00								
***232***	6.41		1.33								
***234***	1.28	2.10	0.67								
***236***	5.13		1.33								

Allele frequencies for drone fathers from each population (Arnot Forest, Apiary 1, and Apiary 2) for 10 variable microsatellite loci. Unique alleles for each population are underlined.

## Discussion

Feral colonies of honey bees, especially ones living more or less isolated from managed colonies, provide insights into the basic ecology of honey bees that cannot be gained from their managed counterparts. This study of the feral honey bee colonies living in the remote Arnot Forest was undertaken to gain insights into their mating biology and to explore how they have been able to survive while the population of managed honey bee colonies in North America has experienced heavy mortality each year and has depended on the aid of beekeepers to survive.

Numerous studies have investigated the mating frequencies of *Apis mellifera* queens living in managed colonies crowded in apiaries (e.g., [29,32]). However, little is known about the mating frequencies of queens of European descent living in feral colonies dispersed across the landscape. Tilley and Oldroyd [37] analyzed three colonies in Australia that were abandoned and thus left unmanaged for several years, although their goal was to compare the paternity distributions of workers and queens rather than the mating frequency of the putatively feral queens. Nielsen [38] used RAPD markers to directly identify worker paternity in six feral colonies of European descent in California. Taken together, these two studies suggest that the queens in feral honey bee colonies of European descent have relatively low mating frequencies (6.6 ± 3.0) compared to queens in managed colonies of European descent (10.1 ± 3.6). In the southern United States, there is also a thriving population of colonies of African descent, and their queens are known to have among the highest mating frequencies in the species (20.0 ± 8.5; [31]). The fact that our estimate of queen mating frequency for the feral Arnot Forest colonies (17.4 ± 9.8) is not different from our estimates of queen mating frequency for the two nearest groups of managed colonies (20.8 ± 5.9, and 16.0 ± 6.4), suggests that the previous estimates for feral colonies of European descent are underestimates because of low allelic diversity [38], small marker number [37], or small colony sample size. Whatever the reason, our results show that queen honey bees in dispersed, feral colonies do not necessarily mate with significantly fewer males than queens in crowded, managed colonies.

It might seem surprising that the low density of honey bee colonies in the Arnot Forest (approximately one colony per square kilometer) does not result in a decreased level of polyandry of the queens in these colonies. This result is, however, consistent with the experiences of queen producers and is understandable given the reproductive biology of honey bees. For example, Neumann et al. [29] examined the mating frequencies of queens at various commercial mating sites in Europe, some of them isolated on islands or in mountain valleys. They found significant differences among some sites, but they found no association between number of nearby drone-source colonies (a proxy for drone abundance) and queen mating frequency. These results, combined with the results reported here, suggest that drone availability is generally not a significant constraint on the mating frequency of honey bee queens. A typical colony raises hundreds if not thousands of drones for each queen that it rears [39], thus the numerical sex ratio is extremely male-biased (even though there is a 1:1 investment into the two sexes because of the resources required to raise a queen and issue a swarm; [40]). Thus, even in regions with low densities of colonies, honey bee queens evidently encounter plentiful drones. Because queens and drones mate in discrete sites (“drone congregation areas”; [41]), the low density of colonies in a feral population does not necessarily create low drone densities where matings occur.

It would be interesting to estimate the density of the honey bee colonies in the Arnot Forest using the techniques proposed by Arundel et al. [42]. To do so, one could mate 10+ queens in the Arnot Forest and quantify the number of drone fathers from distinct colonies among the worker offspring. In the current study, however, we estimated the mating frequencies of the queens in the Arnot Forest population itself, rather than of queens of known pedigrees outside of the Arnot Forest population. Moreover, the frequency of null alleles and the lack of allelic diversity at particular loci rendered us unable to conduct a sufficient pedigree analysis within and among the 10 feral colonies. We were also unable to use the molecular data to infer the effective population size of the feral bees in our study [43] because our sample size was just 10 colonies, too low for the analytical tools currently available.

Previous research on feral populations of honey bees has focused on population genetics, trying to determine what level of genetic difference exists between the feral and managed colonies in a region. Delaney et al. [44] have shown that the genetics of the feral colonies and the managed colonies nearby can be distinct (see also Seeley et al., in press). In the present study, for example, the queens from Apiary 1 were from a single commercial source, and it is apparent from the number of unique drone alleles that these colonies were not immediately introducing a significant number of novel alleles into the surrounding feral population. This separation in genetic identity is even more apparent when comparing pairwise F_st_ analysis between individuals from the Arnot Forest and the two managed apiaries. As such, we can conclude that the queens of the feral colonies in the Arnot Forest rely considerably, although not exclusively, on the drones produced by other feral colonies in this population.

Our findings support the null hypothesis that honey bee queens in feral colonies mate at the same frequency as those in managed colonies. Therefore, our evidence does not support the hypothesis that the population of feral colonies living in the Arnot Forest is able to persist despite the known infestation with *Varroa destructor* [13] and its associated viruses because of heightened levels of polyandry by their queens. Prior studies that have demonstrated strong effects of high intracolonial genetic diversity on resistance to devastating infections have generally tested the effects of extreme differences in queen mating frequency (i.e., single-mating vs. multiple-mating) rather than the differences that are typical of the normal variation in mating frequency (e.g., [45,46]). Neumann and Moritz [47] looked at the correlation between *Varroa* infestation level and queen mating frequency across a typical range of queen mating frequencies and found no significant association. The fact that even a rather low level of polyandry (≥7 mates) strongly enhances a colony’s phenotype suggests that there is some threshold benefit of polyandry above which there are diminishing returns to additional genetic diversity, at least with respect to resisting parasites and pathogens (c.f., [48]).

Exceptionally high levels of queen polyandry are universal among species of the honey bee genus *Apis* [17]. The results of our comparison of feral and managed honey bees in *Apis mellifera* support the view that it is unlikely that the mean mating frequency of queens is a labile trait than can rapidly evolve to adapt to ecological challenges. Franck et al. [49] compared two subspecies in different habitats in Africa and found differences in mating frequency between them, but these populations probably diverged long ago and therefore have experienced protracted selection for higher or lower mating frequencies. The results reported here, which found no difference in mating frequency of queens in the feral colonies in the Arnot Forest vs. queens in the nearest managed colonies outside this forest, supports the notion that mating frequency of queen honey bees is shaped by natural selection acting on an evolutionary, not an ecological, timescale.

## References

[1] Ruttner F (1988) Biogeography and Taxonomy of Honeybees In: Springer V, editor. New York.

[2] Engel MS, Hinojosa-Diaz IA, Rasnitsyn AP (2009) A honey bee from the Miocene of Nevada and the biogeography of Apis (Hymenoptera: Apidae: Apini). Proceedings of the California Academy of Sciences 60: 23–38.

[3] Williams GR, Tarpy DR, Vanengelsdorp D, Chauzat MP, Cox-Foster DL, et al (2010) Colony Collapse Disorder in context. Bioessays 32: 845–846. 10.1002/bies.201000075 20730842 PMC3034041

[4] Chauzat MP, Faucon JP, Martel AC, Lachaize J, Cougoule N, et al (2006) A survey of pesticide residues in pollen loads collected by honey bees in France. Journal of Economic Entomology 99: 253–262. 16686121 10.1603/0022-0493-99.2.253

[5] Chen Y, Evans JD, Smith IB, Pettis JS (2008) Nosema ceranae is a long-present and wide-spread microsporidian infection of the European honey bee (Apis mellifera) in the United States. Journal of Invertebrate Pathology 97: 186–188. 17880997 10.1016/j.jip.2007.07.010

[6] Cox-Foster DL, Conlan S, Holmes EC, Palacios G, Evans JD, et al (2007) A metagenomic survey of microbes in honey bee colony collapse disorder. Science 318: 283–287. 17823314 10.1126/science.1146498

[7] vanEngelsdorp D, Evans JD, Saegerman C, Mullin C, Haubruge E, et al (2009) Colony Collapse Disorder: A Descriptive Study. Plos One 4. 10.1371/journal.pone.0006481 PMC2715894 19649264

[8] Sammataro D, Gerson U, Needham G (2000) Parasitic mites of honey bees: Life history, implications, and impact. Annual Review of Entomology 45: 519–548. 10761588 10.1146/annurev.ento.45.1.519

[9] Korpela S, Aarhus A, Fries I, Hansen H (1992) Varroa jacobsoni Oud in cold climates: population growth, winter mortality and influence on survival of honey bee colonies. Journal of Apicultural Research 31: 157–164.

[10] Kraus B, Page RE Jr. (1995) Population growth of Varroa jacobsoni Oud. in Mediterranean climates of California. Apidologie 26: 149–157.

[11] Oldroyd BP (1999) Coevolution while you wait: Varroa jacobsoni, a new parasite of western honeybees. Trends in Ecology & Evolution 14: 312–315. 10407428 10.1016/s0169-5347(99)01613-4

[12] Magnus R, Szalanski AL (2010) Genetic Evidence for Honey Bees (Apis mellifera L.) of Middle Eastern Lineage in the United States. Sociobiology 55: 285–296.

[13] Seeley TD (2007) Honey bees of the Arnot Forest: a population of feral colonies persisting with Varroa destructor in the northeastern United States. Apidologie 38: 19–29.

[14] Cremer S, Armitage SAO, Schmid-Hempel P (2007) Social immunity. Current Biology 17: R693–R702. 17714663 10.1016/j.cub.2007.06.008

[15] Wilson-Rich N, Spivak M, Fefferman NH, Starks PT (2009) Genetic, individual, and group facilitation of disease resistance in insect docieties. Annual Review of Entomology 54: 405–423. 10.1146/annurev.ento.53.103106.093301 18793100

[16] Strassmann JE (2001) The rarity of multiple mating by females in the social Hymenoptera. Insectes Sociaux 48: 1–13.

[17] Tarpy DR, Nielsen R, Nielsen DI (2004) A scientific note on the revised estimates of effective paternity frequency in Apis. Insectes Sociaux 51: 203–204.

[18] Palmer KA, Oldroyd BP (2000) Evolution of multiple mating in the genus Apis. Apidologie 31: 235–248.

[19] Mattila HR, Seeley TD (2007) Genetic diversity in honey bee colonies enhances productivity and fitness. Science 317: 362–364. 17641199 10.1126/science.1143046

[20] Oldroyd BP, Rinderer TE, Schwenke JR, Buco SM (1994) Subfamily recognition and task specialisation in honey bees (Apis mellifera L.) (Hymenoptera: Apidae). Behavioral Ecology and Sociobiology 34: 169–173.

[21] Tarpy DR, Page RE Jr. (2001) The curious promiscuity of queen honey bees (Apis mellifera): evolutionary and behavioral mechanisms. Annales Zoologici Fennici 38: 255–265.

[22] Hamilton WD (1987) Kinship, recognition, disease, and intelligence: constraints of social evolution In: Kikkawa J, editor. Animal Societies: Theory and Facts. Tokyo: Japanese Scientific Society Press pp. 81–102.

[23] Sherman PW, Seeley TD, Reeve HK (1988) Parasites, pathogens, and polyandry in social Hymenoptera. American Naturalist 131: 602–610. 10.1086/286127 18811329

[24] Schmid-Hempel P (1998) Parasites in Social Insects. Princeton, N. J.: Princeton University Press. 409 p.

[25] Visscher PK, Seeley TD (1982) Foraging strategy of honey bee colonies in a temperate deciduous forest. Ecology 63: 1790–1801.

[26] Visscher PK, Seeley TD (1989) Bee-Lining As a Research Technique in Ecological Studies of Honey Bees. American-Bee-Journal 129: 536–539.

[27] Delaney DA, Keller JJ, Caren JR, Tarpy DR (2011) The physical, insemination, and reproductive quality of honey bee queens (Apis mellifera). Apidologie 42: 1–13. 10.1093/jncimonographs/lgr006 21672889

[28] Crozier RH, Oldroyd BP, Tay WT, Kaufmann BE, Johnson RN, et al (1997) Molecular advances in understanding social insect population structure. Electrophoresis 18: 1672–1675. 9378143 10.1002/elps.1150180934

[29] Neumann P, Moritz RFA, van Praagh J (1999) Queen mating frequency in different types of honey bee mating apiaries. Journal of Apicultural Research 38: 11–18.

[30] Wattanachaiyingcharoen W, Oldroyd BP, Wongsiri S, Palmer K, Paar R (2003) A scientific note on the mating frequency of Apis dorsata. Apidologie 34: 85–86.

[31] Tarpy DR, Caren JR, Delaney DA, Sammataro D, Finley J, et al (2010) Mating frequencies of Africanized honey bees in the south western USA. Journal of Apicultural Research 49: 302–310.

[32] Tarpy DR, Keller JJ, Caren JR, Delaney DA (2012) Assessing the mating 'health' of commercial honey bee queens. Journal of Economic Entomology 105: 20–25. 22420250 10.1603/ec11276

[33] Wang JL (2004) Sibship reconstruction from genetic data with typing errors. Genetics 166: 1963–1979. 15126412 10.1534/genetics.166.4.1963 PMC1470831

[34] Nielsen R, Tarpy DR, Reeve HK (2003) Estimating effective paternity number in social insects and the effective number of alleles in a population. Molecular Ecology 12: 3157–3164. 14629394 10.1046/j.1365-294x.2003.01994.x

[35] Park S (2001) Excel Microsatellite Toolkit Version 3.1.1. Dublin, Ireland: Animal Genomics Lab, University College.

[36] Boomsma JJ, Ratnieks FL (1996) Paternity in eusocial Hymenoptera. Philosophical Transactions of the Royal Society of London, B 351: 947–975.

[37] Tilley CA, Oldroyd BP (1997) Unequal subfamily proportions among honey bee queen and worker brood. Animal Behaviour 54: 1483–1490. 9521799 10.1006/anbe.1997.0546

[38] Nielsen DI (2000) Genetic structure of feral honey bee (Apis mellifera L.) populations in California. Ph.D. Thesis, University of California, Davis. Available: http://search.proquest.com/dissertations/docview/304579935/DDE34018DFF440DAPQ/1?accountid=12725. Accessed 2014 Nov 14.

[39] Seeley TD (2002) The effect of drone comb on a honey bee colony's production of honey. Apidologie-33: 75–86.

[40] Hamilton WD (1975) Gamblers since life began: barnacles, aphids, elms. The Quarterly Review of Biology 50: 175–180.

[41] Loper GM, Wolf WW, Taylor OR (1992) Honey bee drone flyways and congregation areas—radar observations. Journal of the Kansas Entomological Society 65: 223–230.

[42] Arundel J, Oldroyd BP, Winter S (2012) Modelling honey bee queen mating as a measure of feral colony density. Ecological Modelling 247: 48–57.

[43] Moritz RFA, Dietemann V, Crewe R (2008) Determining colony densities in wild honeybee populations (Apis mellifera) with linked microsatellite DNA markers. Journal of Insect Conservation 12: 455–459.

[44] Delaney DA, Meixner MD, Schiff NM, Sheppard WS (2009) Genetic Characterization of Commercial Honey Bee (Hymenoptera: Apidae) Populations in the United States by Using Mitochondrial and Microsatellite Markers. Annals of the Entomological Society of America 102: 666–673.

[45] Seeley TD, Tarpy DR (2007) Queen promiscuity lowers disease within honeybee colonies. Proceedings of the Royal Society B-Biological Sciences 274: 67–72. 17015336 10.1098/rspb.2006.3702 PMC1679871

[46] Tarpy DR (2003) Genetic diversity within honeybee colonies prevents severe infections and promotes colony growth. Proceedings of the Royal Society of London Series B-Biological Sciences 270: 99–103. 12596763 10.1098/rspb.2002.2199 PMC1691209

[47] Neumann P, Moritz RFA (2000) Testing genetic variance hypotheses for the evolution of polyandry in the honeybee (Apis mellifera L.). Insectes Sociaux 47: 271–279.

[48] Tarpy DR, vanEngelsdorp D, Pettis JS (2013) Genetic diversity affects colony survivorship in commercial honey bee colonies. Naturwissenschaften 100: 723–728. 10.1007/s00114-013-1065-y 23728203

[49] Franck P, Koeniger N, Lahner G, Crewe RM, Solignac M (2000) Evolution of extreme polyandry: an estimate of mating frequency in two African honeybee subspecies, Apis mellifea monticola and A.m. scutellata. Insectes Sociaux 47: 364–370.

